# Spontaneous vertebral artery dissection in an elder patient: a case report

**DOI:** 10.11604/pamj.2021.40.219.29825

**Published:** 2021-12-11

**Authors:** Fatima-Ezzahrae Badi, Mouna Sabiri, Samia El Manjra, Samira Lezar, Fatiha Essodegui

**Affiliations:** 1 Department of Central Radiology, Ibn Rochd University Hospital, Casablanca, Morocco

**Keywords:** Vertebrobasilar artery dissection, atherosclerosis, stroke, case report

## Abstract

Vertebrobasilar artery dissection (VBD) is a rare cause of posterior ischemic strokes. It is more likely to occur in young patients with a history of traumatism or hereditary connective tissue disorders. Spontaneous VBD is rare, especially in elder patients. This paper aims to report the case of an old patient that presented with a posterior ischemic stroke due to a spontaneous vertebral artery dissection (SVAD), linked to atherosclerosis. The diagnosis of SVAD was made by Magnetic resonance imaging (MRI) which is the gold standard technique to diagnose this pathology in a short time so the patient could receive adequate treatment.

## Introduction

Dissection of the cervical arteries is a known cause of ischemic vascular accidents; it represents only 0.4% -2.5% of strokes in the general population but 5% -20% of strokes in young people [1]. Magnetic resonance imaging is a sensitive tool for the diagnosis of cervical artery dissections due to the high-resolution imaging and direct visualization of the intramural hematoma. Dissection of the vertebral artery is 3 times less common than dissection of the internal carotid artery. Spontaneous dissections of the vertebral artery in elderly patients are even rarer. We report the case of a dissection of the vertebral artery that occurred in an elderly patient.

## Patient and observation

**Patient information:** a 78-year-old male patient was admitted to the emergency department for swallowing disorders and a brutal left facial paralysis that has progressed for 48 hours. The patient has no particular history apart from chronic smoking and hypercholesterolemia for which the patient is regularly monitored.

**Clinical findings:** on clinical examination, the patient was conscious, apyretic, with a deviation of the left hemiface.

**Timeline of the current episode:** this was the first episode, and have never presented such clinical manifestations. The episode began 48 hours before his admission to the emergency room (ER) by swallowing disorders and brutal left facial paralysis.

**Figure 1:**
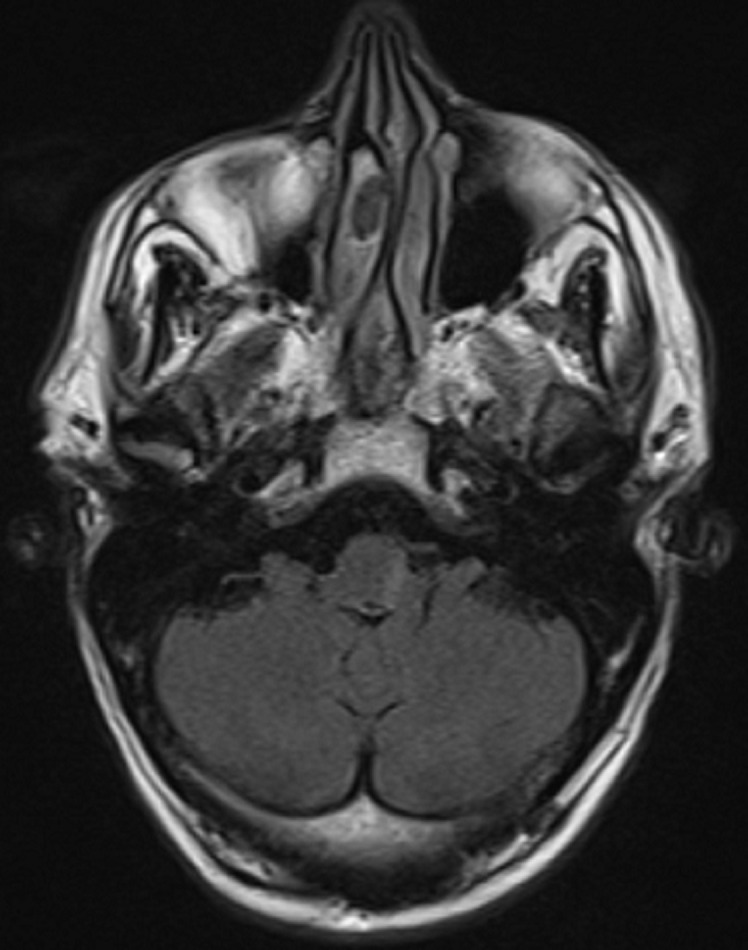
axial flair TSE WI, left bulbar hyperintensity

**Figure 2:**
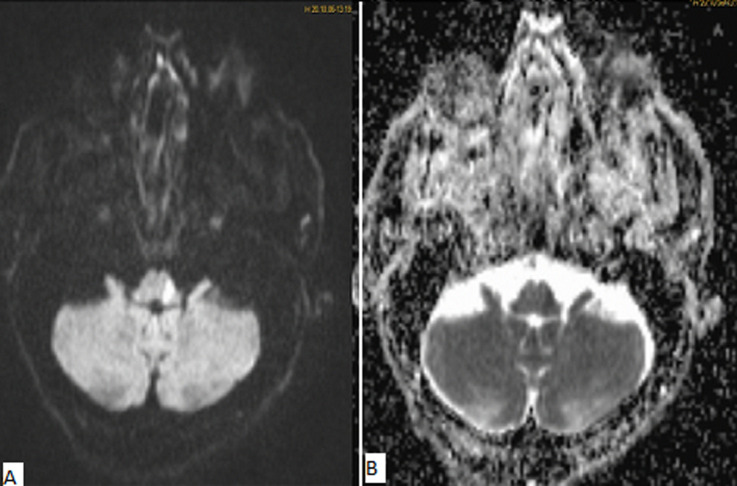
axial diffusion WI with ADC value, showing a restriction of the diffusion with low ADC value

**Figure 3:**
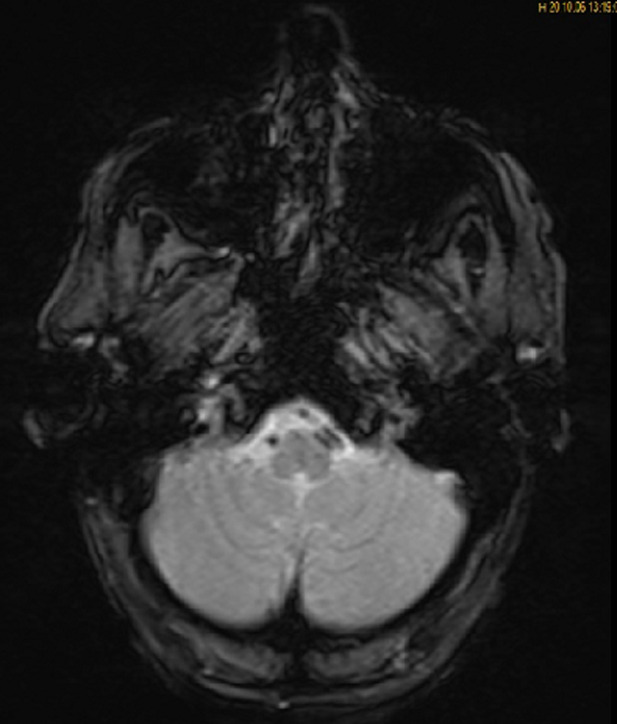
axial T2 GE WI showing left bulbar hypointensity without signal voids

**Figure 4:**
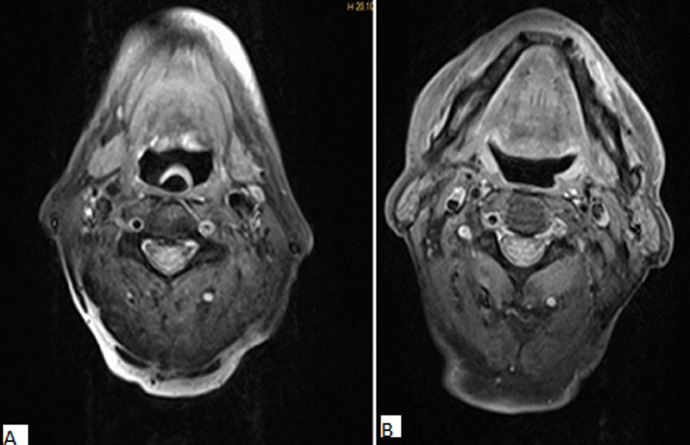
axial T1 fat sat, parietal hematoma of the left vertebral artery, showing a hyperintense signal

**Diagnostic assessment:** a brain MRI was demanded in our radiology department. The MRI was performed on a 1.5T machine, using a stroke standard protocol: i) flair weighted images (WI); ii) diffusion-weighted images (DWI) with ADC values; iii) T2 gradient-echo (GE) weighted images (WI); iv)arterial angiographic sequences (TOF); v) T1 fat-saturated (FS) Turbo spin echo weighted images (TSE WI). The MRI showed a subacute left bulbar ischemic lesion with discreet hyperintense signal on FLAIR WI (Figure 1), and restricted signal on diffusion (DWI) (Figure 2). The T2 GE WI showed a hyperintense signal without void signal (Figure 3). On the arterial angiographic sequence, we note the absence of flow of the left vertebral artery, and a parietal hematoma of the left vertebral artery, showing a hyperintense signal in FS (fat saturated) T1 TSE WI (Figure 4).

**Diagnosis:** the diagnosis of spontaneous dissection of the left vertebral artery was established.

**Therapeutic interventions:** the patient received medical symptomatic treatment and was put on antiplatelet drug and was addressed to physiotherapy and speech therapy.

**Follow-up and outcome of interventions:** after weeks of therapeutic care, the patient was able to do his daily activities and recovered his speech.

**Patient perspective:** he was satisfied with the diagnostic and therapeutic care.

## Discussion

Vertebral artery dissection results from a rip of the intima of the vertebral artery with the introduction of blood between the media and the intima of the vessel resulting from an intramural hematoma which consequently obstructs the vascular lumen responsible for a decrease or absence of flow to the brain. The etiopathogenesis of dissections of vertebrobasilar arteries is not yet fully understood. Multiple risk factors have been identified, whether acquired or constitutional. A history of cervical trauma is common but not universal, hypertension, use of oral contraceptives, migraine, or underlying vascular disease are implicated. Hereditary connective tissue disorders such as Ehlers-Danlos syndrome, Marfan syndrome, alpha1 antitrypsin deficiency, may predispose to arterial dissection. Spontaneous dissection of the vertebral artery most often occurs in its extradural portions. The location of the dissection may determine the clinical manifestations and suggests further management [2]. These spontaneous Vertebral artery dissections (SVAD) can be due to ulceration of an atheromatous plaque in the vessel leading to disruption of the internal elastic lamina and may extend to media leading to the formation of an intramural hematoma that can reduce the lumen of the artery or occlude it. This hypothesis is the same as the one explaining atherosclerotic dissection in the aorta that was confirmed on pathological studies [3].

Conventional angiography has been used for so many years to make the diagnosis of VBD and was the gold standard of VBD exploration. It may demonstrate focal dilatation, proximal or distal stenosis, or fusiform aneurysmal dilatation depending on whether there´s dilation of the artery or stenosis [4]. Recently magnetic resonance angiogram (MRA) and MRI were largely used to explore VBD and this is due to the fact that the intramural hematoma can be directly visualized as an eccentric image narrowing the lumen of the VB artery, with hyperintense signal in T1 weighted images with fat suppression (FS) due to Methemoglobin that constitutes the intraluminal hematoma. The intensity of the signal may vary according to the age of hematoma and reduces when it becomes chronic. The absence of intraluminal flow voids in T2 suggests total occlusion of the VB artery. Sometimes atherosclerotic plaques associated with severe intimal disease could mimic MRI imaging findings in VBD [5]. Brain computed tomography scan is an important technique in the absence of MRA or when MRI cannot be done (MRI contraindications). It is an essential technique scan in emergency allowing the diagnosis of posterior fossa ischemic strokes or subarachnoid hemorrhage. It may sometimes show a characteristic “double lumen” appearance. Cerebral computed tomography (CT) angiography makes it possible to diagnose dissections of the vertebral artery by visualizing the intramural hematoma as a thickened wall, especially with coronal, sagittal reformats, and virtual rendering (VR), demonstrating irregularity of the lumen, as well as make thickening of the arterial wall more easily appreciable. The strength of this case report is to demonstrate the occurrence of artery dissection in a patient with atherosclerosis without a traumatic or other connective tissue diseases, although it is not enough to make atherosclerosis a real risk factor for arteries dissection. It also demonstrates the role of MRA in diagnosing arteries dissection whenever it occurred.

## Conclusion

Our case report reminds physicians that VBD can occur in old and young patients, and should not be dismissed whenever there´s an ischemic stroke of the posterior fossa. This case supports the theory of atherosclerosis as a risk factor of spontaneous vertebral artery dissection. Magnetic resonance angiogram is the gold standard technique to establish the diagnosis and might be useful to predict individual risk of occurrence of ischemic strokes.
